# Analysis of the Initiating Events in HIV-1 Particle Assembly and Genome Packaging

**DOI:** 10.1371/journal.ppat.1001200

**Published:** 2010-11-18

**Authors:** Sebla B. Kutluay, Paul D. Bieniasz

**Affiliations:** 1 Aaron Diamond AIDS Research Center, Laboratory of Retrovirology, The Rockefeller University, New York, New York, United States of America; 2 Howard Hughes Medical Institute, The Rockefeller University, New York, New York, United States of America; Northwestern University, United States of America

## Abstract

HIV-1 Gag drives a number of events during the genesis of virions and is the only viral protein required for the assembly of virus-like particles in vitro and in cells. Although a reasonable understanding of the processes that accompany the later stages of HIV-1 assembly has accrued, events that occur at the initiation of assembly are less well defined. In this regard, important uncertainties include where in the cell Gag first multimerizes and interacts with the viral RNA, and whether Gag-RNA interaction requires or induces Gag multimerization in a living cell. To address these questions, we developed assays in which protein crosslinking and RNA/protein co-immunoprecipitation were coupled with membrane flotation analyses in transfected or infected cells. We found that interaction between Gag and viral RNA occurred in the cytoplasm and was independent of the ability of Gag to localize to the plasma membrane. However, Gag:RNA binding was stabilized by the C-terminal domain (CTD) of capsid (CA), which participates in Gag-Gag interactions. We also found that Gag was present as monomers and low-order multimers (e.g. dimers) but did not form higher-order multimers in the cytoplasm. Rather, high-order multimers formed only at the plasma membrane and required the presence of a membrane-binding signal, but not a Gag domain (the CA-CTD) that is essential for complete particle assembly. Finally, sequential RNA-immunoprecipitation assays indicated that at least a fraction of Gag molecules can form multimers on viral genomes in the cytoplasm. Taken together, our results suggest that HIV-1 particle assembly is initiated by the interaction between Gag and viral RNA in the cytoplasm and that this initial Gag-RNA encounter involves Gag monomers or low order multimers. These interactions *per se* do not induce or require high-order Gag multimerization in the cytoplasm. Instead, membrane interactions are necessary for higher order Gag multimerization and subsequent particle assembly in cells.

## Author Summary

Human immunodeficiency virus (HIV) assembles at the plasma membrane of the infected host cell, resulting in the release of infectious virus particles. HIV assembly is directed by the viral structural protein, Gag that performs a number of functions including specific recruitment of viral genomic RNA and multimerization around this RNA to form a virus particle. However, it is currently not clear where in the cell these two key events, Gag-RNA interaction and Gag multimerization, are initiated and whether they are coordinated. In this study we provide strong evidence that recruitment of viral genomic RNA by Gag is initiated in the cytoplasm of the host cell. However, this interaction *per se* does not require or induce a high degree of Gag multimerization, as Gag is present as monomers or dimers in the cytoplasm. In contrast, plasma membrane seems to be the only site at which higher order Gag multimerization occurs. Notably, at least a fraction of the Gag dimers in the cytoplasm are bound to the viral RNA. These results provide deeper insights to our understanding of the molecular details of the initiating events in HIV-1 assembly, which are potential targets for development of new antiviral drugs.

## Introduction

Assembly of human immunodeficiency virus type 1 (HIV-1) is a multi-step process that is driven and coordinated by the viral Gag protein. During assembly, Gag molecules selectively recruit unspliced viral genomic RNA for packaging into virions from a large pool of cellular RNA molecules. The Gag:RNA interaction is mediated by the nucleocapsid (NC) domain of Gag that binds directly to the packaging sequence (psi), which is composed of four stem loops located within the 5′ UTR and the 5′ end of the *gag* gene [1], [2], [3], [4]. Another key event is the recruitment of Gag molecules to the plasma membrane, the major site for productive HIV-1 assembly [5], [6], [7]. Plasma membrane targeting is directed by functions in the matrix (MA) domain, consisting of the N-terminal myristoyl group [8], [9] as well as a cluster of basic amino acids [10] and other residues that confer specific recognition of PI(3,4)P2 [11], [12], [13]. Subsequent steps in HIV-1 assembly include the oligomerization of a few thousand Gag molecules around a nucleating core Gag:RNA complex at the plasma membrane, a process driven by the capsid (CA) and NC domains of Gag [14], [15], [16], [17]. The final step is the recruitment of cellular factors that enable virion budding by the C-terminal p6 domain [18], [19], [20]. After the release of particles from the cell surface, a number of proteolytic cleavage events in Gag lead to major structural changes, yielding infectious virions.

Gag multimerization is obviously a key event in HIV-1 assembly and multiple domains of Gag, including MA, CA and NC, have been proposed to play a role in this process. Although biochemical and structural studies [21] indicate that recombinant MA can form trimers, MA is not likely to be the driving force for Gag multimerization, as Gag proteins lacking most or all of MA can assemble into virus particles [22], [23], [24], [25]. Conversely, the role of CA and NC in Gag multimerization and accurate particle assembly has been substantiated in a variety of experimental settings. In particular, mutations affecting the C-terminal domain (CTD) of CA disrupt assembly [26], [27], [28], [29], a finding supported by structural data [30]. Additionally, although it is not required for the early stages of virion assembly [31], the N-terminal domain (NTD) of CA mediates CA multimerization in mature virions through the formation of CA hexamers [26], [28], [29], [32], [33]. A number of studies have indicated that NC contributes to Gag multimerization, mainly by virtue of its basic residues that are important for RNA binding [34], [35], [36], [37]. It is likely that NC mediates Gag-Gag interactions indirectly, through recruitment of viral RNA that plays a structural role in virions [38] by serving as a scaffold for Gag multimerization. This view is supported by the finding that addition of RNA promotes particle assembly in vitro [39], [40], [41], whereas removal of RNA results in disruption of particles [38], [39]. Likewise, RNase treatment of recombinant Gag [35] or Gag/Gag-Pol complexes [42] inhibits Gag-Gag interaction.

The notion that viral RNA plays an important role in HIV-1 assembly raises two important and related questions about early events in the process. The first is where in the cell Gag initially interacts with viral RNA. Two potential sites are the cytoplasm and plasma membrane. Initiation of RNA packaging at the plasma membrane would require the viral RNA and Gag to move separately to this location. Recent live-cell imaging studies indicate that viral genomes that are otherwise highly dynamic in the cytoplasm become anchored at the plasma membrane in the presence of Gag, before particle assembly is detectable [43]. Therefore, it is possible that a small number of Gag molecules bind to the viral RNA in the cytoplasm and bring it to the plasma membrane, or that RNA binds to a small number of Gag molecules that are already situated at the plasma membrane. Currently, there is no available data that would support or distinguish between either hypothesis.

The second key question about the initiating steps in particle assembly is whether Gag forms oligomers in the cytoplasm prior to membrane binding. Techniques that rely on epitope masking of Gag upon multimerization [36], as well as biochemical analyses [44], [45], [46], [47], have shown that efficient localization to plasma membrane correlates with the ability of Gag to undergo the multimerization that accompanies assembly. Additionally, a number of studies using fluorescence resonance energy transfer (FRET) assays have also indicated that Gag multimers form primarily at the plasma membrane [48], [49], [50], [51], [52]. Detection of Gag multimers in the cytoplasm has been more controversial, perhaps due to a modest degree of multimerization and experimental limitations (e.g. background fluorescence in FRET assays). Still, a number of FRET [49], [51], [52] and bimolecular fluorescence complementation (BiFC) assays have suggested that some level of Gag multimerization occurs in the cytoplasm [53], [54]. However, none of these assays can address precisely the extent and stoichiometry of Gag multimerization in the cytoplasm of cells.

Interestingly, in vitro biochemical assays have shown that recombinant Gag exists in monomer-dimer or monomer-trimer equilibrium depending on buffer conditions, particularly the presence of inositol phosphates that might mimic the presence of membrane [55]. Whether Gag exists in a similar type of equilibrium in the cytoplasm and whether it binds RNA as monomers or multimers in cells is still largely unknown, although there is some in vitro evidence obtained using truncated Gag proteins suggests that it might bind RNA as a dimer [56].

In this study, we aimed to elucidate the initial site of Gag-RNA interaction and whether Gag forms multimers, perhaps on viral RNA, before recruitment to the plasma membrane. To accomplish this, we developed assays in which we combined subcellular fractionation with RNA-immunoprecipitation (RNA-IP) and/or covalent protein crosslinking. Our results indicate that Gag-RNA interaction takes place in the cytoplasm both in transiently transfected and in infected cells. This interaction was not affected by the ability of Gag to localize to the plasma membrane, strongly suggesting that the initial site of Gag-RNA interaction is the cytoplasm. However, absence of CA-CTD led to a decrease in immunoprecipitable Gag-RNA complexes in the cytoplasm and, particularly, at the plasma membrane, suggesting that proper multimerization of Gag might be important for stabilizing Gag-RNA interactions. Crosslinking/subcellular fractionation analyses of Gag molecules in cells showed that Gag forms high-order multimers exclusively at the plasma membrane. In contrast, Gag appeared predominantly as monomers, but did form low-order multimers, in the cytoplasm. We tested whether these low-order multimers form on viral RNA using a sequential RNA-IP assay, which suggested that at least a fraction of Gag molecules multimerize on a given viral genome. Taken together, our results suggest that Gag-RNA interaction initially takes place in the cytoplasm and involves Gag monomers or low-order multimers. Moreover, Gag-RNA interaction *per se* does not induce or require higher order Gag multimerization in the cytoplasm. Instead, membrane interactions appear to be required to induce higher order Gag multimerization in cells.

## Results

### Immunoprecipitation assay to assess HIV-1 Gag:RNA interaction

The development of live-cell imaging techniques has allowed the visualization of individual virion assembly events that take place at the plasma membrane [43], [57]. These studies have shown that viral RNA, that is otherwise highly mobile, can become anchored at the plasma membrane in the presence of Gag. Nonetheless, Gag only becomes visible at the site of the membrane-anchored RNA at later times, suggesting that a small number of Gag molecules are initially responsible for retaining viral RNA at the plasma membrane [43]. Because the initial interaction between Gag and RNA apparently involves an invisible number of Gag molecules, visual techniques are limited in their ability to analyze earlier assembly events, i.e. whether the initial Gag-RNA interaction occurs in the cytoplasm, or at the plasma membrane.

**Figure 1:**
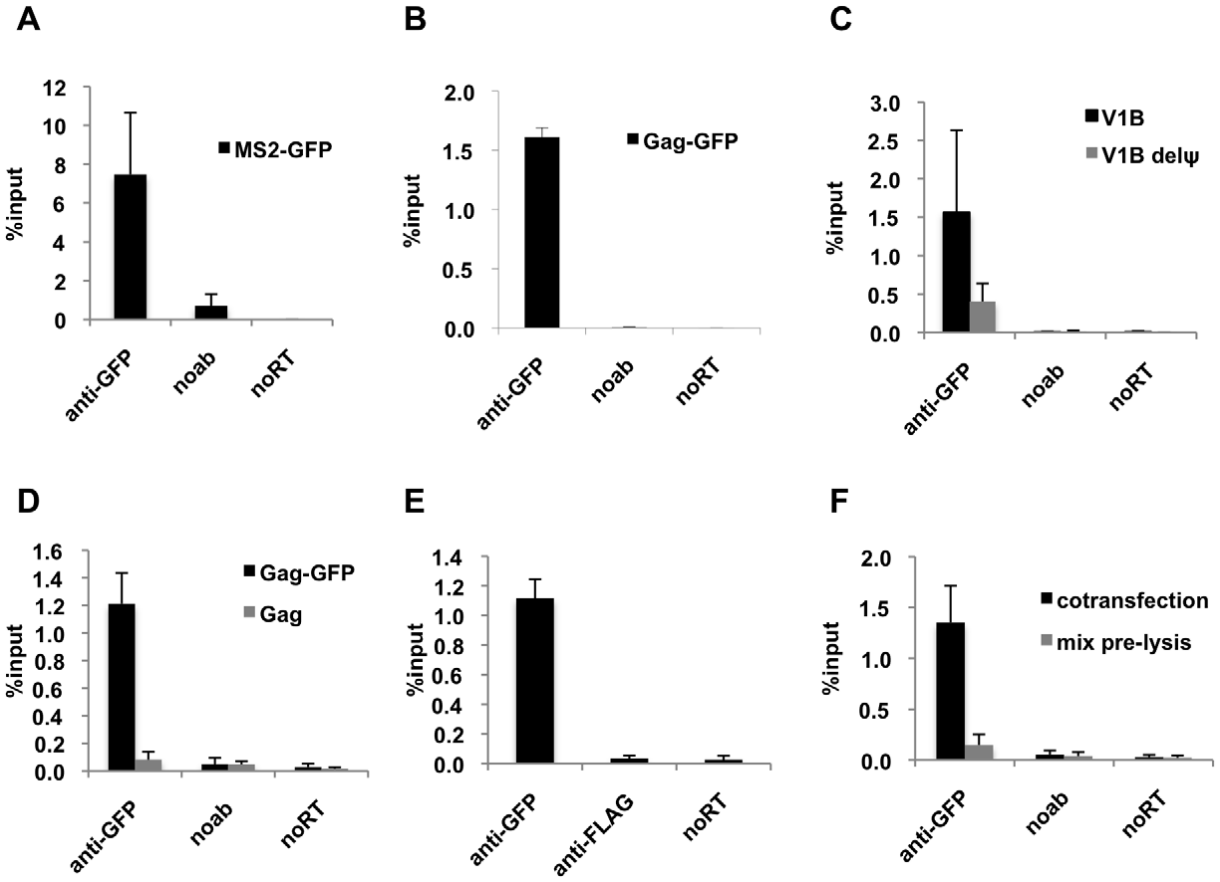
Efficient immunoprecipitation of HIV-1 genomes by MS2-GFP or Gag-GFP. (**A and B**) Lysates of 293T cells coexpressing V1B-MS2 RNA and either MS2-GFP (A) or Gag-GFP (B) were prepared at 24-h post-transfection. RNA-protein complexes were immunoprecipitated using anti-GFP antibodies. Immunoprecipitations without antibodies (noab) were carried in parallel. Immunoprecipitated V1B-MS2 RNA was quantitated by qRT-PCR and is represented as fraction of input RNA prior to immunoprecipitation (% input). (**C**) RNA-IP assay was performed as in (B) where the viral genome was either V1B or V1BΔΨ. (**D**) RNA-IP assay in 293T cells coexpressing V1B-MS2 viral RNA and Gag-GFP or untagged Gag. (**E**) RNA-IP assay was performed as in (B) but an immunoprecipitation using anti-FLAG antibodies was included as negative control. (**F**) 293T cells separately expressing Gag-GFP or V1B-MS2 viral RNA were mixed before cell lysis followed by immunoprecipitation (gray bars). RNA-IP assay was then performed as in (B). Immunoprecipitations from 293T cells that were cotransfected with Gag-GFP and V1B-MS2 plasmids were carried in parallel (black bars). Data in (A-F) represents the average of two independent experiments, where error bars indicate the range between the averages of two experiments. RNA extracted from 10% of the cell lysate that was used in immunoprecipitation was subjected to qRT-PCR without reverse transcription (noRT) to control for the presence of contaminating viral DNA.

To address this issue, we developed a quantitative RNA-IP assay where protein-RNA complexes are immunoprecipitated and the amount of RNA that is bound to the protein of interest is analyzed by qRT-PCR. Since coexpression of a packageable HIV-1 genomic RNA carrying binding sites for the bacteriophage MS2 coat protein (V1B-MS2) and the MS2-GFP fusion protein allowed the successful visualization of viral RNA:protein complexes [43], we first tested whether a complex of V1B-MS2 RNA and MS2-GFP protein could be immunoprecipitated. Cells (293T) transiently transfected with V1B-MS2 RNA and MS2-GFP expression plasmids were processed for RNA-IP assay at 24 hours post-transfection. As shown in Figure 1A, about 8% of the total viral RNA in the cell lysate was immunoprecipitated by anti-GFP antibodies. We then tested whether HIV-1 Gag-GFP could similarly be used to immunoprecipitate a complex containing the V1B-MS2 RNA. Indeed, V1B-MS2 RNA could also be efficiently immunoprecipitated in a complex with Gag-GFP (Figure 1B). The lower efficiency of RNA-IP with Gag-GFP (1 to 2% of the total RNA in the cell lysate) as compared to with MS2-GFP may be due to the presence of multiple copies of high affinity binding sites on viral RNA for the latter protein. Additionally, it is important to note that the fraction of the input viral RNA that is immunoprecipitated is determined by the fraction of the viral RNA that is actually associated with Gag, not simply the efficiency with which these complexes are immunoprecipitated. One would expect that, in cells, not all genomic RNA molecules will be associated with Gag and that not all Gag molecules will be associated with genomic RNA. This, combined with the fact that immunoprecipitation of a given protein is rarely 100% efficient, explains why only a proportion of the viral RNA that is present in cells is recovered by immunoprecipitation of the Gag protein.

Next, we analyzed the specificity of the RNA-IP assay. First, Gag-GFP was coexpressed with either the V1B-MS2 viral RNA or a mutant derivative, V1B-Δ ψ-MS2, in which stem loops 3 and 4 of the packaging signal were deleted. Association of V1B-Δ ψ-MS2 RNA with Gag was reduced by about 3 to 4-fold as compared to V1B-MS2 RNA (Figure 1C). This data correlates with the previously observed reduction in virion infectivity and RNA packaging associated with the V1B-Δ ψ-MS2 viral RNA [43]. To test the specificity of anti-GFP immunoprecipitation, we performed parallel immunoprecipitations using untagged Gag (Figure 1D) or an irrelevant antibody, anti-FLAG (Figure 1E). As expected, viral RNA was not immunoprecipitated using the untagged Gag or by anti-FLAG antibodies (Figure 1D, E).

We then tested whether the immunoprecipitated Gag-RNA complexes form in the living cell prior to lysis, or whether they form as an artifact of cell lysis and subsequent experimental steps. Thus, 293T cells separately expressing V1B-MS2 RNA and Gag-GFP were mixed before cell lysis and processed for RNA-IP in parallel with cells coexpressing both viral RNA and Gag-GFP. As indicated in Figure 1F, Gag-GFP was unable to immunoprecipitate the V1B-MS2 when the RNA and protein were expressed in separate cells that were mixed before cell lysis. These results indicate that the immunoprecipitated Gag-RNA complexes form in the cell and that there is little, if any, association of Gag with viral RNA after cell lysis. Taken together these results strongly suggest that our RNA-IP assay is specific and represents biologically relevant RNA:protein interaction events that occur in living cells.

### Interaction of HIV-1 Gag with the viral genome is increased by CA but does not require Gag localization to membranes

**Figure 2:**
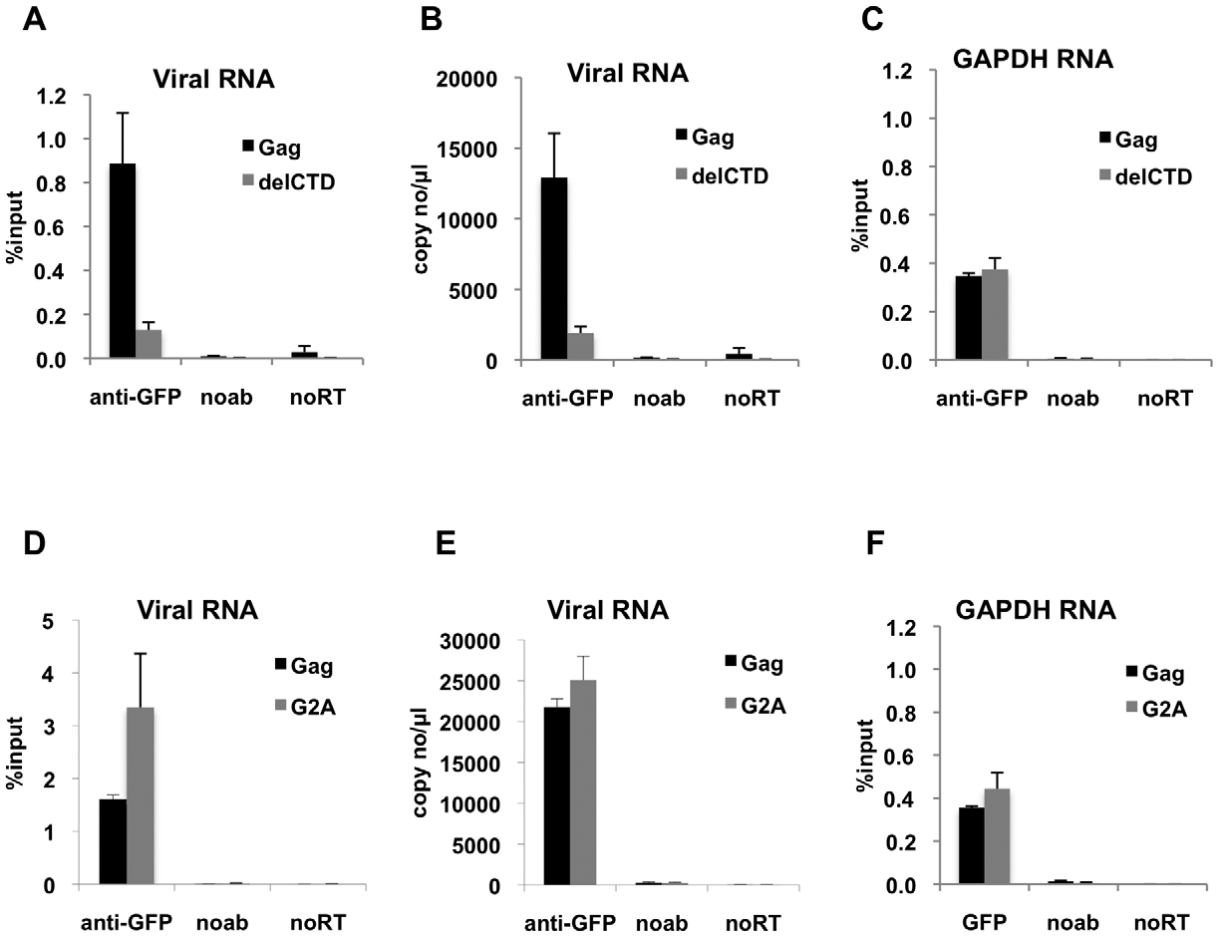
Interaction of Gag with HIV-1 genome is enhanced by an intact CA domain but not by Gag myristoylation. (**A-C**) V1B-MS2 viral RNA bound to either Gag-GFP (black bars) or Gag-delCTD-GFP (gray bars) was immunoprecipitated by anti-GFP antibodies from lysates of 293T cells. Immunoprecipitations without antibodies (noab) were included in parallel as controls. (**D-F**) V1B-MS2 viral RNA bound to either Gag-GFP (black bars) or Gag-G2A-GFP (gray bars) was immunoprecipitated by anti-GFP antibodies as in (A-C). Immunoprecipitated RNA was quantitated by qRT-PCR to detect viral RNA (A, B, D, E) or cellular GAPDH RNA (C, F) as described in Materials & Methods and is represented either as fraction of input material prior to immunoprecipitation (% input) or copies of DNA per µl of reverse transcribed template (copy no/µl). Data represents the average of two independent experiments, where error bars indicate the range between the averages of two experiments. noRT =  as explained in legend to Figure 1.

Because HIV-1 assembly requires an intact CA-CTD, and given the possibility that proper Gag multimerization might affect RNA binding, we performed RNA-IP assays using lysates of 293T cells coexpressing V1B-MS2 RNA and a mutated version of Gag-GFP that lacks the CTD of CA (Gag-delCTD-GFP). Notably, about 5-fold less V1B-MS2 was immunoprecipitated by Gag-delCTD, as compared to WT Gag, in terms of the fraction of the input RNA (Figure 2A) or the absolute numbers of copies of RNA (Figure 2B). In contrast, immunoprecipitation of cellular GAPDH RNA by Gag-GFP was not affected by the presence or absence of CA-CTD (Figure 2C). It should be noted that immunoprecipitation of GAPDH RNA by Gag is expected, as HIV-1 particles can package cellular mRNAs, usually in proportion to their cellular abundance [38], [58]. Interaction of Gag with GAPDH RNA, however, was less efficient than with viral RNA (compare the percentage of the input RNA that was immunoprecipitated in Figure 2A and 2C).

Next, we determined whether an HIV-1 Gag mutant that should not localize to the plasma membrane could still interact with viral RNA. Mutation of the N-terminal glycine residue of MA to alanine (G2A) blocks the attachment of a myristoyl group to Gag and hence prevents its recruitment to the plasma membrane [8], [9]. Notably, G2A-Gag, unlike WT Gag, did not affect the behavior of GFP-labeled viral RNA in cells [43], suggesting the possibility that Gag membrane localization might be important for RNA-Gag interactions. Interestingly, G2A-Gag-GFP immunoprecipitated the V1B-MS2 RNA with an efficiency that was equal to or higher than that of WT Gag (Figure 2D, E). This result indicates that membrane binding is not required for the association of Gag with the viral genome and suggests that the Gag-RNA interaction is initiated in the cytoplasm. Like Gag-delCTD, G2A-Gag also immunoprecipitated GAPDH RNA at a similar efficiency to that of WT Gag (Figure 2F). Taken together, these results suggest that viral genome packaging is initiated by Gag:RNA interactions in the cytoplasm and that Gag:RNA interactions are, in part, dependent on the presence of an intact CA-CTD.

### Analysis of HIV-1 Gag interactions with viral genomes in subcellular fractions

To analyze more directly whether Gag interacts with viral RNA in the cytoplasm, we coupled the RNA-IP assay to membrane flotation analyses. Lysates of 293T cells transiently expressing Gag-GFP, G2A-Gag-GFP or Gag-delCTD-GFP together with V1B-MS2 were separated using membrane flotation gradients and ten 1 ml fractions of the gradient were collected. In this assay, membranes and associated proteins concentrated mainly in fraction 3, whereas the cytoplasmic content remained in fractions 9 and 10. We isolated total RNA and proteins from each fraction and analyzed each for the presence of the viral and cellular RNA as well as Gag proteins. Additionally, RNA-IP assays were performed on fractions 3 (membranes) and 10 (cytoplasm).

**Figure 3:**
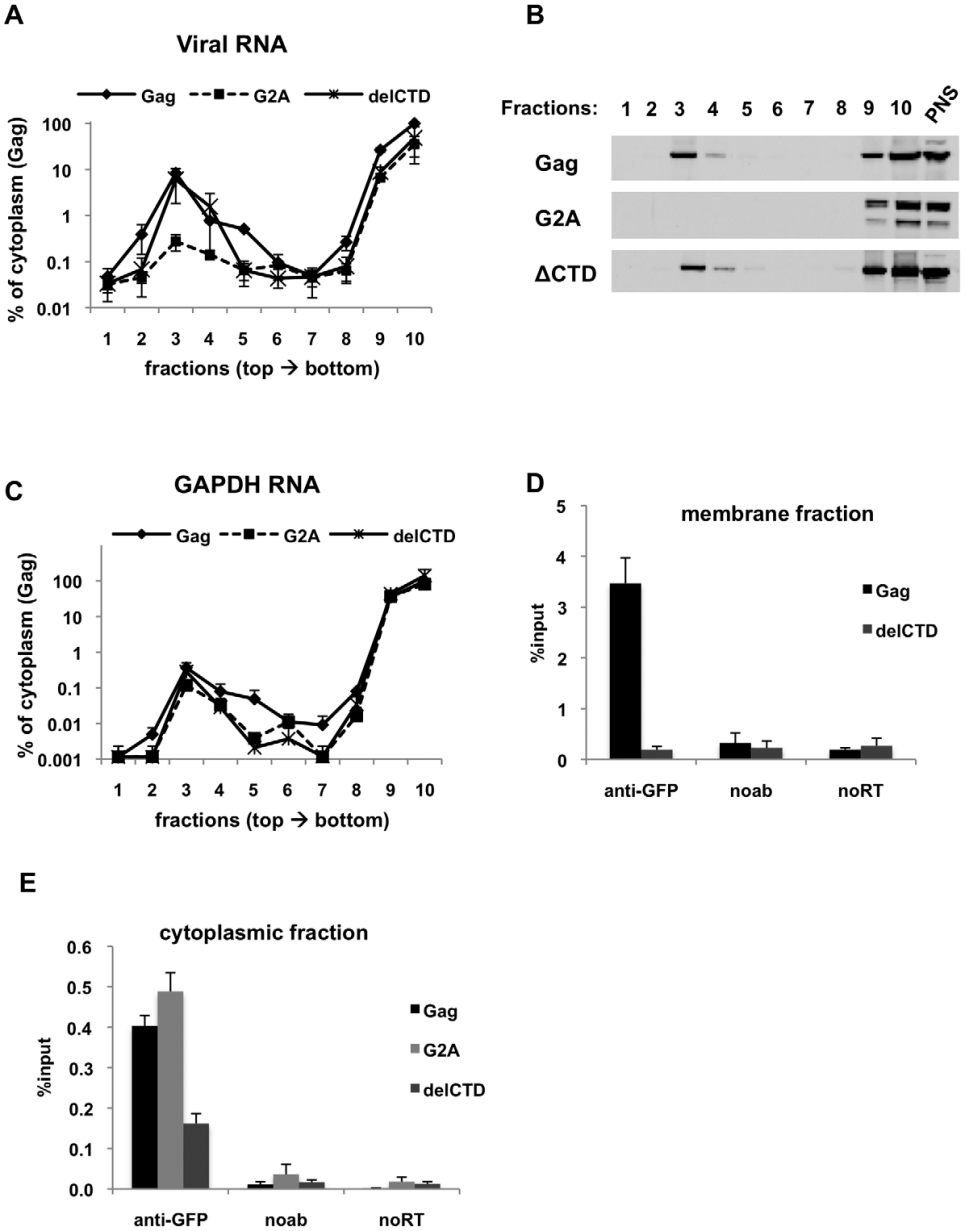
Immunoprecipitation of HIV genomic RNA from membrane and cytoplasmic fractions by Gag, G2A-Gag and Gag-delCTD. (**A-E**) 293T cells coexpressing V1B-MS2 and either Gag-GFP, G2A-Gag-GFP or Gag-ΔCTD-GFP were processed for subcellular fractionation at 24-h post-transfection. Ten fractions from the top of the membrane flotation gradient were collected. Total RNA and proteins from these fractions were isolated as explained in Materials & Methods. (**A and C**) V1B-MS2 (A) and GAPDH (C) RNA in fractions were quantitated by qRT-PCR. Data is represented as the relative number of copies of cDNA with as compared to the tenth, cytoplasmic fraction of Gag-GFP sample that was arbitrarily set to a value of 100%. (**B**) Western blot analysis of Gag-GFP, G2A-Gag-GFP and Gag-ΔCTD-GFP in sucrose fractions and in post-nuclear supernatant (PNS) using anti-HIV-1 MA antibodies. (**D and E**) After subcellular fractionation, immunoprecipitations from the membrane (D) and cytoplasmic (E) fractions were performed using anti-GFP antibodies as explained in Materials & Methods. Parallel immunoprecipitations were carried in the absence of antibodies (noab). Immunoprecipitated V1B-MS2 RNA is quantitated by qRT-PCR and is represented as a fraction of input material prior to immunoprecipitation (% input, [D, E]). Data in (A, C, D, E) represents the average of two independent experiments where error bars indicate the range. noRT =  as explained in legend to Figure 1.

In the presence of WT Gag-GFP, viral RNA and Gag-GFP localized to two peaks in the gradient corresponding to the membrane and cytoplasmic fractions (Figure 3A, 3B). Surprisingly, even though Gag-delCTD-GFP was less efficient than WT Gag-GFP in immunoprecipitating viral RNA from total cell lysates (Figure 2A, B), it was fully capable of recruiting viral RNA to the membrane fraction (Figure 3A). As expected, there was significantly less viral RNA in the membrane fraction in the presence of G2A-Gag-GFP (Figure 3A), which did not itself appear in the membrane fractions (Figure 3B). These results suggest that recruitment of viral RNA to the plasma membrane is independent of CA-mediated Gag multimerization but is simply a function of Gag membrane localization. In contrast to viral RNA, the presence of a relatively low level of GAPDH RNA in the membrane fraction was unaffected by Gag or mutant versions thereof (Figure 3C). The presence of GAPDH RNA in the plasma membrane fraction likely represents nonspecific association of RNA and membranes, perhaps as a result of resealing of membranous vesicles following dounce homogenization. This idea is supported by the finding that a similarly low level of V1B-MS2 RNA in the membrane fraction was observed even in the absence of Gag (data not shown). It should be emphasized that there were approximately 300–400 fold lower concentrations of GAPDH RNA in the membrane fraction as compared to the cytoplasmic fraction (Figure 3C). In contrast, in the presence of WT Gag-GFP, viral RNA was present in the membrane fraction at about 10% of the concentration in the cytoplasm fraction (Figure 3A).

The presence of Gag-RNA complexes at the plasma membrane and in the cytoplasm were examined by performing RNA-IP assays using fractions 3 and 10 of the membrane flotation gradients, respectively. As expected, WT Gag was able to efficiently coprecipitate viral RNA from the membrane fraction (Figure 3D). Surprisingly, even though Gag-delCTD-GFP could localize to the membrane fraction (Figure 3B) and recruit viral RNA to this fraction as efficiently as WT Gag (Figure 3A), it was not able to efficiently coprecipitate viral RNA from this fraction (Figure 3D). It is likely that Gag-delCTD forms a complex with RNA that is insufficiently stable to effectively survive the immunoprecipitation procedure. This idea is consistent with previous imaging studies in which Gag-delCTD was observed to be diffusely distributed at the plasma membrane and that some RNA molecules anchored at the plasma membrane by Gag-delCTD were found to dissociate after a few minutes [43]. As expected, RNA-IP from the membrane fraction of cells expressing G2A-Gag did not yield detectable viral RNA as this protein does not localize and recruit viral RNA to the plasma membrane (data not shown).

In parallel with our findings using total cell lysates (Figure 2), Gag-GFP and G2A-Gag-GFP coprecipitated viral RNA with almost equal efficiencies from the cytoplasmic fraction, but the ability of Gag-delCTD to immunoprecipitate viral RNA appeared comparatively diminished (Figure 3E). Thus, these results indicate that Gag-viral RNA complexes can form in the cytoplasm and that their stability is, in part, dependent on the presence of intact CA. We emphasize that the Gag-RNA complexes analyzed in this assay do not form as a consequence of cell lysis and further manipulations but represent events that take place in the cell. Evidence for this is provided by mixing experiments where cells separately expressing Gag-GFP and V1B-MS2 RNA were mixed before cell lysis and processed for membrane flotation and RNA-IP from cytoplasmic fractions in parallel with cells coexpressing Gag-GFP and viral RNA. Similar to our observations using total cell lysates (Figure 1F), mixing cells that separately express Gag-GFP and viral RNA before cell lysis did not yield detectable Gag-RNA complexes in the membrane and cytoplasmic fractions (data not shown).

### Gag interacts with viral RNA in the cytoplasm of HIV-1 infected cells

**Figure 4:**
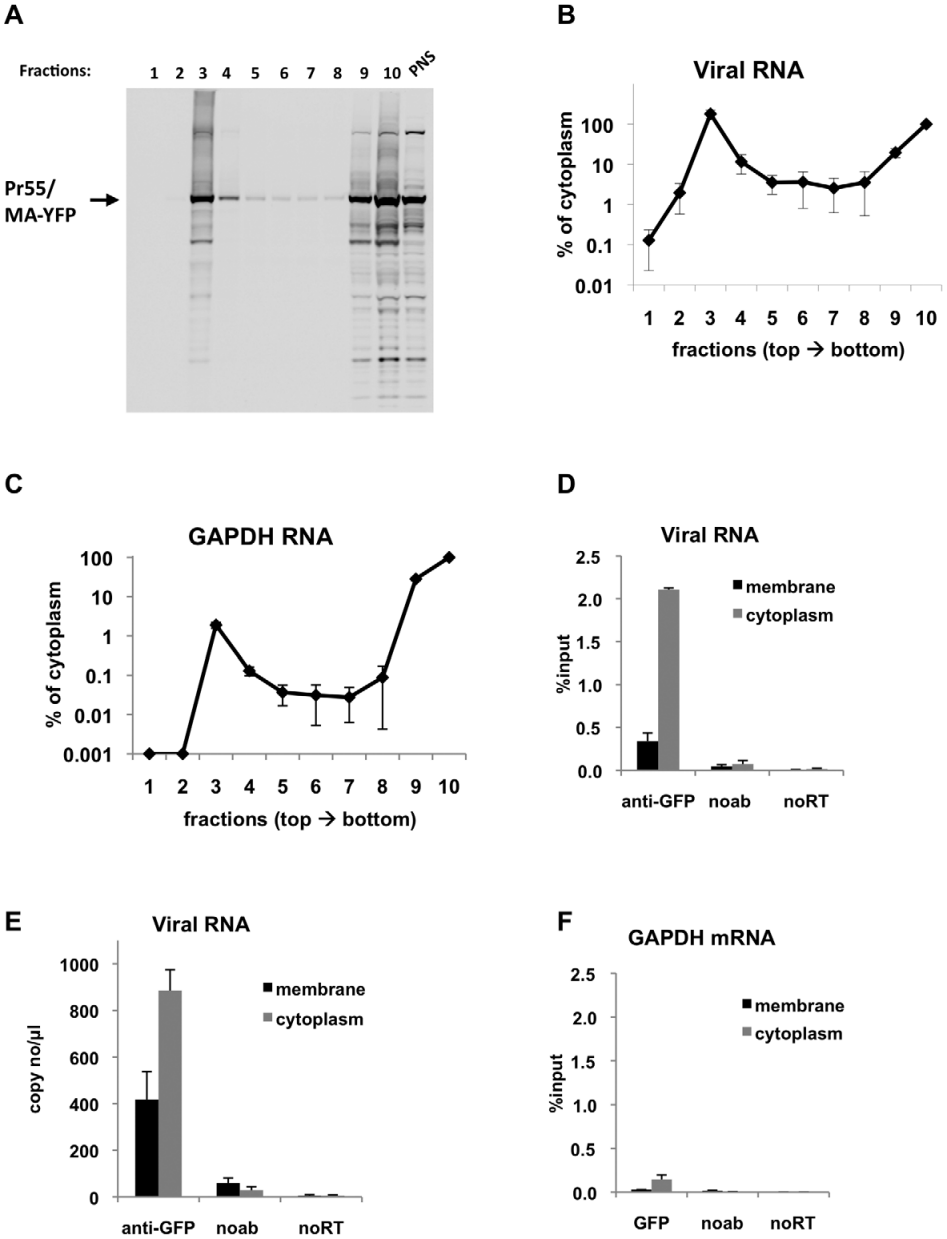
Immunoprecipitation of genomic viral RNA from membrane and cytoplasmic fractions of HIV-1 infected MT2 cells. (**A-F**) MT2 cells were infected with VSV-G pseudotyped NL4-3 MA-YFP/PR- at MOI = 1 and processed for subcellular fractionation at 48-h post-infection. Ten fractions from the top of the membrane flotation gradient were collected. Total RNA and proteins from these fractions were isolated as explained in Materials & Methods. (**A**) Western blot analysis of MA-YFP Gag in sucrose fractions and in post-nuclear supernatant (PNS) using anti-HIV-1 CA antibodies. (**B and C**) Viral (B) and GAPDH (C) RNA in fractions was quantitated by qRT-PCR. Data is represented as the relative number of copies of cDNA as compared to the tenth, cytoplasmic fraction sample that was arbitrarily set to a value of 100%. (**D-F**) Immunoprecipitations from the membrane (black bars) and cytoplasmic (gray bars) fractions were performed using anti-GFP antibodies. Parallel immunoprecipitations without antibodies (noab) were included as controls. Immunoprecipitated viral RNA (D and E) or GAPDH RNA (F) was quantitated by qRT-PCR and is represented as either fraction of input material prior to immunoprecipitation (% input) or number of copies of cDNA per µl of PCR template. Data in (B-F) represents the average of two independent experiments where error bars indicate the range. noRT =  as explained in legend to Figure 1.

In order to examine the interaction of Gag with viral RNA in a more physiologically relevant context, we repeated the membrane flotation/RNA-IP experiments in infected cells. Specifically, MT2 cells were infected with an HIV-1 derivative carrying the immunoprecipitation tag (YFP) in the stalk region of MA and an inactive protease (NL4-3 MA-YFP/PR-). This defective genome was introduced into MT2 cells using VSV-G pseudotyped virions that were generated with an excess of complementing WT HIV-1 GagPol proteins. Thus, the virions were infectious for only a single cycle and the infected MT2 cells expressed, *de novo*, the YFP-tagged, PR-defective Gag and GagPol proteins. At 48 h after infection, these infected cells were fractionated by membrane flotation and processed as above. As expected, the Gag-MA-YFP protein was present in both the membrane and the cytoplasmic fractions (Figure 4A). Similarly the viral RNA was present in two peaks in the gradient corresponding to the membrane and cytoplasmic fractions (Figure 4B). Compared with transfected 293T cells (Figure 3A), recruitment of viral RNA to the plasma membrane appeared more efficient in infected cells, as indicated by the similar concentrations viral RNA in membrane and cytoplasmic fractions (Figure 4B). This may be due to a more optimal balance of virion RNA and Gag protein levels in infected as opposed to transfected cells. As expected, GAPDH RNA was present at much lower levels in the plasma membrane fraction compared with the cytoplasmic fraction of infected MT2 cells (Figure 4C), suggesting that the membrane-associated viral RNA represents specific recruitment by Gag.

Next, viral RNA-Gag complexes were immunoprecipitated from the membrane and cytoplasmic fractions of infected MT2 cells. In agreement with transient transfection based assays, Gag-RNA complexes were successfully isolated from both the cytoplasmic and membrane fractions (Figure 4D, E). The efficiency of immunoprecipitation for viral RNA in the membrane fraction was lower than the cytoplasmic fraction (Figure 4D, E), even though these fractions contained almost equal amounts of viral RNA prior to immunoprecipitation (Figure 4B). This may be due to inaccessibility of the YFP immunoprecipitation tag at the plasma membrane where Gag multimerization is likely to be extensive. Indeed, Gag epitope occlusion during particle assembly has been observed before by others [36]. Immunoprecipitation of GAPDH mRNA was much less efficient than viral RNA in both the membrane and cytoplasmic fractions (Figure 4F) in parallel with experiments using transfected cell lysates (Figure 2C, F). Nevertheless, in support of our findings using transfection-based assays, these results clearly show that immunoprecipitable Gag-RNA complexes form in the cytoplasm of HIV-1 infected cells.

### HIV-1 Gag can from low order multimers in the cytoplasm but requires membrane binding for high order multimerization in cells

The above results strongly suggest that Gag can bind to viral RNA while in the cytoplasm. A number of in vitro [55], [56] and in vivo [49], [51], [52], [53], [54] findings have suggested that Gag multimerization might be initiated in the cytoplasm of cells, perhaps triggered by RNA binding. However, none of these assays could address the extent or stoichiometry of Gag multimerization in cells. The fact that Gag, G2A-Gag and Gag-delCTD immunoprecipitate viral RNA at different efficiencies prompted us to analyze whether these proteins could form multimers in the cytoplasm of cells, and how the extent of multimerization in the cytoplasm compared with that at the plasma membrane. We used a chemical crosslinking approach in which 293T cells coexpressing Gag, G2A-Gag or Gag-delCTD and viral RNA were crosslinked by EGS, a membrane permeable crosslinker, and then analyzed using membrane flotation assays. Proteins from membrane and cytoplasmic fractions were precipitated, delipidated and analyzed in parallel with post-nuclear supernatants and mock-crosslinked samples by western blotting.

**Figure 5:**
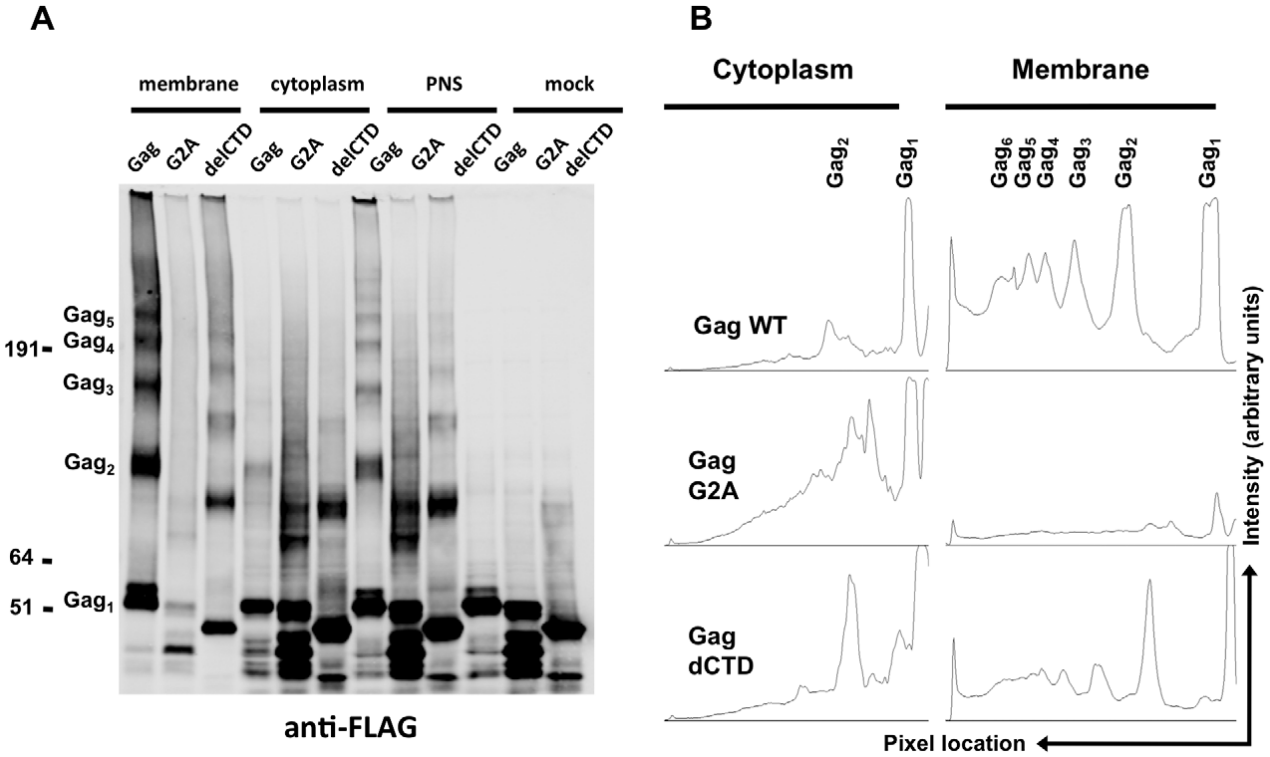
Multimerization of Gag, G2A-Gag, Gag-delCTD in plasma membrane and cytoplasm. 293T cells coexpressing FLAG and HA-tagged Gag or its derivatives (G2A-Gag or Gag-delCTD) together with V1B-MS2 viral RNA were cross-linked by treatment with 1mM EGS and used in membrane flotation assay. (**A**) Western blot analysis of Gag, G2A-Gag and Gag-delCTD in membrane fractions (lanes 1–3), cytoplasmic fractions (lanes 4–6), post-nuclear supernatants (PNS, lanes 7–9) and in mock crosslinked post-nuclear supernatants (lanes 10–12) using mouse anti-FLAG antibodies. (**B**) Quantitative analysis of membrane (lanes 1–3) and cytoplasmic (lanes 4–6) fractions of the western blot in (A). *x*-axis shows the pixel location and *y*-axis indicates the average pixel intensity in the lane that is analyzed. Degree of Gag multimerization is marked by subscripts (i.e. Gag_3_ corresponds to Gag trimers).

The majority of the WT Gag protein was crosslinked and clearly formed high-order multimers at the plasma membrane (Figure 5A, B). A fraction of Gag was present as monomers (Figure 5A, B), perhaps due either to incomplete crosslinking by EGS or incomplete assembly. Notably, Gag-delCTD, which does not assemble into particles, also formed high-order multimers at the plasma membrane (Figure 5A, lane 3), however the extent of this multimerization was significantly reduced as compared to WT Gag (Figure 5A, B). Higher order multimerization of Gag-delCTD was somewhat unexpected given that CA-CTD participates in Gag-Gag interactions and is essential for particle assembly. Presumably other domains of Gag, such as MA, the CA-NTD and/or NC, are in contact with each other and can be crosslinked by EGS leading to the observed pattern of Gag-delCTD multimers.

Notably, none of the Gag proteins formed high-order multimers in the cytoplasm, although cytoplasmic low-order Gag multimers (i.e. dimers) were apparent in each case (Figure 5A, lanes 4, 5, 6). A minor caveat is that the G2A Gag protein appeared to be somewhat prone to breakdown; thus the smeared appearance of G2A-Gag dimers in the cytoplasm is likely due to multimerization between intact Gag-G2A and its degradation products (Figure 5A, lane 5). Nonetheless, G2A-Gag and Gag-delCTD dimers were as or more abundant than were WT Gag dimers in the cytoplasm (Figure 5B). Taken together, these results indicate that Gag forms only low order multimers in the cytoplasm and that the different RNA binding abilities of Gag mutants cannot be simply explained by the extent to which they multimerize in the cytoplasm. These results also suggest that high-order Gag multimers are neither required for, nor induced upon, binding of Gag to viral RNA in the cytoplasm. Rather, high order Gag multimerization requires Gag to bind to membrane.

### Gag can form multimers on viral RNA in the cytoplasm

Since we observed that a fraction of Gag forms low-order multimers in the cytoplasm, we directly tested whether viral RNA was associated with multimeric, cytoplasmic Gag. Specifically, we performed a sequential RNA-IP (seq-RNA-IP) assay in 293T cells co-expressing FLAG- and HA-tagged Gag proteins together with viral RNA. After dual crosslinking by EGS and formaldehyde, total cell lysates were used in immunoprecipitation assays using anti-HA or anti-FLAG antibodies. The eluates from these first immunoprecipitations were then used in a second round of immunoprecipitation using anti-FLAG or anti-HA antibodies, respectively, and the viral RNA content of first and second round immunoprecipitations was evaluated by qRT-PCR.

**Figure 6:**
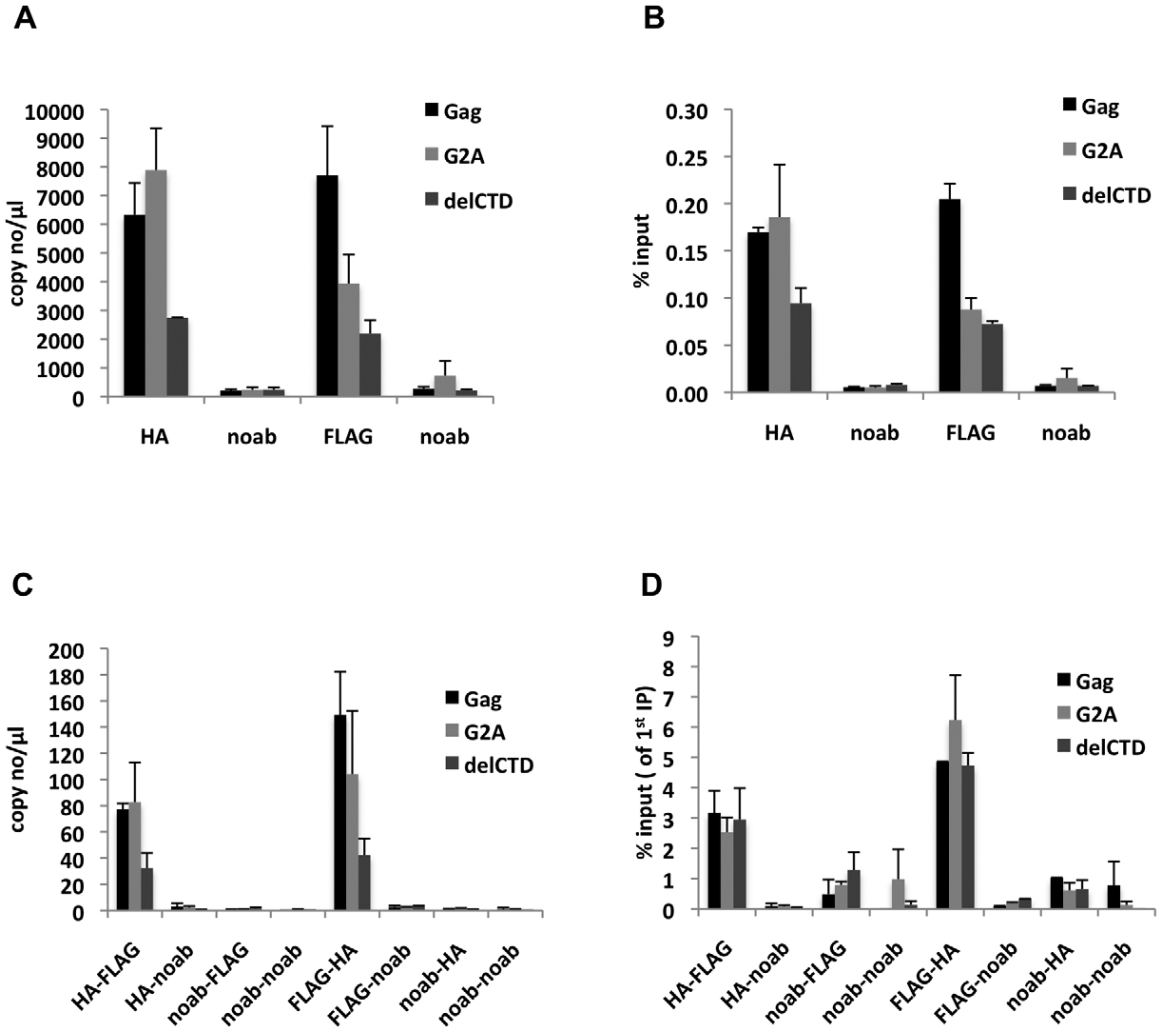
Gag molecules form multimers on viral RNA. 293T cells coexpressing FLAG and HA-tagged Gag or its derivatives (G2A-Gag or Gag-delCTD) together with V1B-MS2 viral RNA were used in seq-RNA-IP analysis after crosslinking with EGS and formaldehyde. (**A, B**) Viral RNA that was eluted after the first immunoprecipitation with anti-HA or anti-FLAG antibodies, or in the absence of antibodies (noab) is quantitated by qRT-PCR as in previous figures. (**C, D**) RNA-protein complexes obtained from the first immunoprecipitation (A, B) were used in a subsequent immunoprecipitation. Order of immunoprecipitations is indicated on the *x*-axis. Data is represented as either number of copies of cDNA per µl of PCR template (A, C) or fraction of input material (% input, B, D). Data in (A-D) shows the average of two independent experiments where error bars indicate the range.

Analyses of the first immunoprecipitations indicated that WT Gag and G2A-Gag immunoprecipitated viral RNA at similar levels, whereas Gag-delCTD exhibited a decreased level of viral RNA coprecipitation (Figure 6A, B). This result is in general agreement with RNA-IP results from cell lysates in the absence of crosslinking (Figure 2), but it should be noted that these immunoprecipitations yielded a smaller fraction of the input RNA (∼0.1–0.2% of the input RNA was detected in a single round of immunoprecipitation) likely due to the fact that RNA chemical crosslinking and milder elution conditions were employed.

When the initial immunoprecipitation with anti-HA antibody was followed by a second immunoprecipitation with an anti-FLAG antibody, viral RNA was easily detected after a second round of immunoprecipitations, regardless of which Gag protein was used (Figure 6C). Similar results were obtained when the order of immunoprecipitation was reversed (HA followed by FLAG or FLAG followed by HA, Figure 6C). Interestingly, when the second round seq-RNA-IP efficiencies were compared, Gag, G2A-Gag and Gag-delCTD all appeared a to be present as multimers on the viral RNA at similar levels (Figure 6D). Specifically. if two different proteins simultaneously associate with a given RNA, then the expected outcome in a seq-RNA-IP assay is to observe a significantly higher enrichment of RNA during the second immunoprecipitation, as opposed to the first [59]. Note, however, that even if all viral RNA molecules are bound by Gag dimers, not all Gag-HA molecules will multimerize with Gag-FLAG molecules on the viral RNA. Rather, there will be a distribution of homodimeric (HA-HA or FLAG-FLAG) and heterodimeric (HA-FLAG) Gag molecules on the viral RNA. Importantly, the fraction of RNA that was immunoprecipitated during the second round of immunoprecipitation was significantly enhanced (∼20-fold) in both HA-FLAG and FLAG-HA seq-RNA-IPs (compare values in Figure 6B and 6D). This result strongly suggests that Gag forms multimers in association with at least a fraction of viral RNA.

Notably, even though seq-RNA-IP assays were done on total cell lysates, the G2A-Gag mutant that cannot localize to the plasma membrane (Figure 3, 5) exhibited a similar degree of multimerization on viral RNA in the seq-RNA-IP assay as did the WT Gag protein. This finding suggests that the detection of viral RNA in the sequential immunoprecipitation was primarily a result of Gag-RNA binding events that occurred in the cytoplasm. We therefore suggest that at least a fraction of the low-order Gag multimers detected in the cytoplasm (Figure 5) were bound to viral RNA.

## Discussion

Given the comparative absence of information about the events that initiate HIV-1 assembly in living cells, we adopted a variety of strategies to analyze this process in cells. First, we developed a quantitative RNA-IP assay that was sensitive, specific and represented Gag-RNA interactions that take place in cells. By coupling this assay to membrane flotation analyses, we were able to show that HIV-1 Gag-RNA complexes form in the cytoplasm of both transiently transfected and infected cells. The fact that formation of these complexes was independent of the ability of Gag to localize to membranes suggests that the cytoplasm is the site where HIV-1 RNA packaging is initiated. Interestingly, the Gag protein of Rous sarcoma virus (RSV) was shown to require nuclear localization for efficient genome packaging [60], [61], whereas the Gag protein of feline immunodeficiency virus was shown to colocalize with viral RNA at the nuclear envelope [62]. Although our results do not exclude the possibility that Gag-RNA interactions are initiated in the nucleus, the presence of little, if any, Gag in the nucleus at steady-state [6], [57], [62] makes it difficult to perform biochemical analyses that could reveal such events, if they were to occur. Nonetheless, the ability of HIV-1 RNA to be packaged requires it to be exported from the nucleus, but the precise export pathway taken by the RNA does not appear to be critical [63]. This suggests that RNA packaging by Gag is initiated in the cytoplasm. A remaining question is whether there are distinct locations in the cytoplasm where HIV-1 Gag interacts with viral RNA or whether Gag-RNA interaction is a stochastic process, where diffusing Gag and RNA molecules form stable complexes randomly as they encounter each other. The mobility of both Gag and viral RNA in the cytoplasm [43], [64] and the apparently rather non-selective interaction of Gag with cellular RNAs [58] is more consistent with the latter hypothesis.

The stability of Gag-RNA complexes both in the cytoplasm and plasma membrane seemed to depend on the CTD of CA, as evidenced by the reduced ability of Gag-delCTD to immunoprecipitate viral RNA. It is likely that the Gag-delCTD/RNA complexes disintegrate during immunoprecipitation, rather than fail to form efficiently in the cell, as Gag-delCTD was fully capable of recruiting viral RNA to the plasma membrane. This finding is in agreement with previous imaging experiments, which revealed that some viral RNA molecules recruited by Gag-delCTD dissociate from the plasma membrane [43]. Given that Gag-delCTD formed multimers at the plasma membrane less extensively than WT Gag, these results might also suggest a role for higher-order Gag multimerization in stabilizing Gag-RNA interactions at the plasma membrane. Alternatively, it is possible that the conformation of Gag in the context of a multimer is influenced by the CA-CTD so as to increase its intrinsic RNA binding affinity – perhaps CA-CTD:CA-CTD contacts optimally position the linked NC domains to better recognize the viral RNA psi sequence.

Even though a number of FRET and BiFC studies indicated that plasma membrane is the major site of Gag multimerization, there has been inconsistency in the published conclusions about whether and to what extent HIV-1 Gag forms multimers in the cytoplasm [48], [49], [50], [51], [52], [53], [54]. Our results are the first to show that a fraction of Gag in the cytoplasm appeared as dimers, but we were not able to detect higher-order Gag multimers in the cytoplasm. Of note, higher levels of G2A-Gag and Gag-delCTD dimers were detected in the cytoplasm as compared to WT Gag. A possible explanation for this observation could be that soon after binding to the viral RNA as low-order multimers, the WT Gag-RNA complexes acquire higher affinity for, or move to, the plasma membrane, where further oligomerization takes place. Thus, a block in membrane binding (in G2A-Gag) or a decrease in the stability of Gag-RNA interactions (in Gag-delCTD) might lead to the accumulation of dimers in the cytoplasm. In contrast to cytoplasmic Gag, plasma membrane-associated Gag molecules formed high order multimers. It was noticeable that Gag-delCTD also formed multimers with apparently similar stoichiometry to WT Gag at the plasma membrane (albeit to lesser extent), even though it is completely incapable of assembling into particles. These multimers are presumably formed by Gag:Gag contacts in MA, CA-NTD or NC domains of Gag. This finding, however, reinforces the finding that localization to the plasma membrane seems to be required for high order Gag multimerization in cells.

Although we cannot exclude the possibility that only a fraction of the Gag dimers detected in the cytoplasm are bound to viral RNA, sequential RNA-IP assays suggest that at least a fraction of Gag molecules form multimers on viral RNA in the cytoplasm. Gag dimerization on viral RNA is likely mediated by RNA sequences around the packaging signal. However, these results do not distinguish between the possibilities that Gag dimers in the cytoplasm form on a single viral RNA molecule or are present on RNA dimers [65]. The latter possibility would require the presence of Gag molecules at a distance of, at the most, 16.1 Å from each other, the spacer arm length of EGS that was used for crosslinking. Currently there is no structural evidence that would favor either of these possibilities over the other.

Overall, our findings suggest that HIV-1 genome packaging is initiated in the cytoplasm. This Gag:RNA interaction does not require or induce high-order Gag multimerization in the cytoplasm, but may involve Gag dimers or low-order multimers, that also form in the cytoplasm. This initial Gag:RNA complex is sufficiently stable to survive immunoprecipitation. Subsequently, Gag-viral RNA complex is recruited to the plasma membrane where high-order Gag multimerization occurs and virion assembly is completed.

## Materials and Methods

### Cell lines, viruses and infections

293T (ATCC #: CRL-11268) cells were obtained from ATCC and grown in DMEM supplemented with 10% fetal bovine serum. MT-2 cells, obtained from the NIH AIDS Research and Reference Reagent Program, were cultured in RPMI supplemented with 10% fetal bovine serum.

VSV-G pseudotyped NL4-3 (MA-YFP/PR-) viruses were produced by transfection of 293T cells with the proviral plasmid, a complementing codon-optimized Gag-Pol expression plasmid and VSV-G expression vector using polyethyleneimine (PolySciences, Warrington, Pennsylvania, United States) as described previously [66]. Virus titers were determined by FACS analysis of target MT-2 cells. Infections were initiated in media containing 5 µg/ml polybrene and input viruses were removed at 6–8 hours post-infection by washing cells several times in complete medium.

### Plasmids

Construction of plasmids expressing codon-optimized HIV-1 Gag-GFP (pCR3.1/HIV-Gag-GFP) [67] and MS2-GFP [43] was described previously. pCR3.1/HIV-Gag-G2A, lacking the myristoylation signal, and pCR3.1/HIV-Gag-dCTD, lacking the C-terminal domain of capsid, were previously described [43], [67] and were used to generate the GFP-tagged versions. For constructing HA- and FLAG-tagged Gag proteins, oligonucleotides coding for three consecutive copies of HA and FLAG tags flanked by NotI and XhoI restriction endonuclease sites were annealed in vitro and cloned into NotI-XhoI digested pCR3.1/HIV-Gag-GFP, G2A-GFP and dCTD-GFP plasmids.

Construction of proviral plasmids V1B, V1B-MS2 and V1B-Δ ψ-MS2 was previously described [43], [68]. NL4-3-derived MA-YFP/PR- proviral plasmid was generated by ligating an AgeI/SpeI fragment of NL4-3/PR- encoding an inactive protease, to AgeI/SpeI-digested NL4-3/MA-YFP plasmid, which incorporates YFP into the stalk region of MA [6].

### Antibodies

Antibodies used in RNA-immunoprecipitation, sequential-RNA-immunoprecipitation and western blot assays were as follows: mouse monoclonal anti-GFP (Roche), mouse monoclonal anti-FLAG (Sigma), mouse monoclonal anti-HA (HA.11 Covance), rabbit polyclonal anti-HIV-1 MA (NIH), mouse monoclonal anti-HIV-1 p24CA (183-H12-5C).

### RNA immunoprecipitation and data analysis

RNA immunoprecipitation (RNA-IP) assay was performed as described previously with minor modifications [69]. Briefly, 1×10^7^ cells were lysed in 250 µl of polysome lysis buffer (10 mM 4-(2-hydroxyethyl)-1-piperazineethanesulfonic acid (HEPES), pH 7.0, 0.1 M potassium chloride, 5 mM magnesium chloride, 25 mM EDTA, and 0.5% Nonidet P-40, 2 mM DTT) supplemented with SuperaseIN (Ambion) and protease inhibitors (Roche). After preclearing with Protein G-sepharose beads (GE Healthcare), lysates were diluted in immunoprecipitation buffer (50 mM Tris-HCl pH 7.4, 150 mM sodium chloride, 1 mM magnesium chloride, 0.05% Nonidet P-40, 1 mM DTT, 15 mM EDTA, supplemented with RNaseOUT). Immunoprecipitations were performed overnight at 4°C in the presence of 5-10 µg of antibody followed by incubation with Protein G-sepharose beads for another 4–5 hours. Parallel immunoprecipitations in the absence of antibody were included as controls. After several washes with NT2 buffer (50 mM Tris-HCl pH 7.4, 150 mM sodium chloride, 1 mM magnesium chloride, 0.05% Nonidet P-40), RNA was eluted by proteinase K (Roche) treatment and purified by phenol:chloroform extraction and ethanol precipitation. RNA was further purified by DNase (Roche) treatment and one more round of phenol:chloroform extraction and ethanol precipitation.

For quantitation of the RNA-IP assay, RNA samples were reverse-transcribed using ImProm-II Reverse Transcription system (Promega). The resulting cDNA was used as template for quantitative real-time PCR (qRT-PCR) using ABI 7500 Fast RT-PCR system. Viral RNA (V1B-MS2 or NL4-3 and its derivatives) was detected by previously described primer-probe pairs [70] and Fast Start TaqMan Probe master mix (Roche). Detection of V1B-MS2ΔΨ and GAPDH RNA was done by a SYBR Green-based assay with the following primer pairs:

V1B-MS2ΔΨ: F: 5′ CAAATGGTACATCAGGCCATATCACCT and R: 5′ TCCTTCTGATAATGCTGAAAACATGGGTAT;

GAPDH: F: 5′ AGGTCATCCCTGAGCTGAAC and R: 5′ GCAATGCCAGCCCCAGCGTC

A standard curve using serial threefold dilutions of input samples (10% to 0.04% of the input) or known copy numbers of NL4-3 plasmid was generated to quantitate the signals from immunoprecipitation samples.

### Sequential RNA immunoprecipitation

For sequential RNA immunoprecipitation (seq-RNA-IP) assays, 1×10^7^ 293T cells were co-transfected with 4 µg of FLAG-tagged Gag, G2A-Gag or Gag-delCTD, 4 µg of HA-tagged Gag, G2A-Gag or delCTD-Gag and 2 µg V1B-MS2 expression plasmids. At 24 hours post-transfection, cells were crosslinked by 1 mM ethylene glycol bis [succinimidylsuccinate] (EGS, Pierce) for 30 min followed by 1% formaldehyde for 10 min at room temperature. Crosslinking was stopped by the addition of 100 mM Tris-Cl and 100 mM glycine. Cells were then washed in 1X PBS three times, resuspended in 500 µl of radioimmunoprecipitation assay buffer (50 mM Tris [pH 7.4], 150 mM NaCl, 1 mM EDTA, 1% Triton X-100, 1% sodium deoxycholate, 0.1% sodium dodecyl sulfate), supplemented with protease and RNase inhibitors and sonicated three times for 20 seconds at power setting 2.5 of a model 550 Sonic Dismembrator (Fisher Scientific). Lysates were precleared by centrifugation and protein-G sepharose bead incubation for 1 hour at 4°C. Immunoprecipitations were performed as in the RNA-IP assay described above using anti-HA and anti-FLAG antibodies. After the first immunoprecipitation, protein-RNA complexes were eluted by a 10-minute incubation of Protein G beads in 150 µl elution buffer (1% SDS, 0.1M NaHCO_3_) at 65°C. Sixty µl of the eluates were then subjected to a second immunoprecipitation. After the second immunoprecipitation, RNA was eluted by Proteinase K treatment. Formaldehyde crosslinking was reversed by incubation at 65°C for two hours and RNA was further purified as in the RNA-IP assay described above.

### Membrane flotation assay (equilibrium flotation centrifugation)

The membrane flotation assay was based on a previously described protocol [71]. In brief, approximately 1×10^7^ cells were washed three times with NTE buffer (100 mM NaCl, 10 mM Tris [pH 7.4], 1 mM EDTA) and resuspended in 500 µl of hypotonic buffer (10 mM Tris [pH 7.4], 1 mM EDTA) supplemented with protease inhibitors. Samples were lysed by dounce homogenization and adjusted to 150 mM NaCl and 1 mM MgCl_2_. Nuclei and intact cells were removed by centrifugation for 10 minutes at 1000× *g*, 4°C. Thereafter, 350 µl of supernatant was mixed with 1650 µl of 90% sucrose solution (prepared in 150 mM NaCl, 10 mM Tris [pH 7.4], 1 mM EDTA, 1 mM MgCl_2_) and overlayed with 6.5 ml of 65% and 2.5 ml of 10% sucrose solutions prepared similarly and supplemented with protease inhibitors. When necessary, RNase inhibitors (RNaseOUT and SuperaseIN) were added to the solutions and buffers listed above. Centrifugation was performed in a Beckman SW41 Ti rotor at 35,000 rpm for 18 h. Ten fractions (1 ml each) were collected from the top of the gradient and used for RNA-IP, RNA extraction or protein analyses by western blotting.

In cases where membrane flotation assay was coupled to the RNA-IP assay, 400 µl of the membrane (3^rd^ fraction) or cytoplasmic fractions (10^th^ fraction) were diluted in 2X immunoprecipitation buffer. The remainder of the immunoprecipitation protocol was carried out as described in the above RNA-IP protocol.

For profiling the viral and cellular RNA, 40 µl of each sucrose fraction was resuspended in 200 µl of the RNA-IP assay elution buffer. RNA was then purified and quantitated as indicated in the RNA-IP protocol.

The remaining material from fractions was precipitated overnight by trichloroacetic acid (TCA) at a final concentration of 10%. Precipitated protein was collected by centrifugation and washed twice with 10% TCA and once with ice-cold acetone. Samples were air-dried briefly and resuspended in SDS-PAGE loading buffer for analysis by western blotting.

### Crosslinking-based Gag multimerization assay

Approximately 1×10^7^ 293T cells, transiently expressing Gag and its derivatives were collected in 1X PBS and crosslinked by treatment with 1 mM EGS, a membrane-permeable crosslinker. After 30-minutes of incubation at room temperature, crosslinking was stopped by addition of Tris-Cl at a final concentration of 100 mM. Cells were then subjected to the membrane flotation assay as indicated above. After TCA precipitation of membrane and cytoplasmic fractions (3^rd^ and 10^th^ fractions from the top of the gradient respectively), samples were resuspended in 9M Urea/2% Triton X-100 and delipidated by methanol:chlorofom extraction [72]. Proteins were separated on NuPAGE 3–8% Tris-Acetate gels (Invitrogen) and transferred to nitrocellulose membranes. Blots were probed with mouse anti-FLAG antibodies, followed by goat anti-mouse antibodies conjugated to IRDye800CW. Fluorescent signals were detected and quantitated using a LICOR Odyssey scanner. ImageJ software (National Institutes of Health, Bethesda, MD) was used for quantitative analysis of Gag multimerization.
